# Role of Silicon in Mitigation of Heavy Metal Stresses in Crop Plants

**DOI:** 10.3390/plants8030071

**Published:** 2019-03-21

**Authors:** Javaid Akhter Bhat, S. M. Shivaraj, Pritam Singh, Devanna B. Navadagi, Durgesh Kumar Tripathi, Prasanta K. Dash, Amolkumar U. Solanke, Humira Sonah, Rupesh Deshmukh

**Affiliations:** 1 Department of Genetics and Plant Breeding, Indian Agricultural Research Institute, New Delhi 110012, India; javid.akhter69@gmail.com; 2 Département de Phytologie, Université Laval, Québec City, QC G1V 0A6, Canada; sraj100@gmail.com; 3 National Agri-Food Biotechnology Institute, Mohali 140306, India; raghav.pritam26dec@gmail.com; 4 National Research Centre on Plant Biotechnology, New Delhi 110012, India; devnova2460@gmail.com (D.B.N.); prasanta01@yahoo.com (P.K.D.); amolsgene@gmail.com (A.U.S.); 5 Amity Institute of Organic Agriculture, Amity University, Uttar Pradesh, Noida 201313, India; dktripathiau@gmail.com

**Keywords:** metal stress, toxicity, silicon, Si-fertilization, genomics, transporter genes

## Abstract

Over the past few decades, heavy metal contamination in soil and water has increased due to anthropogenic activities. The higher exposure of crop plants to heavy metal stress reduces growth and yield, and affect the sustainability of agricultural production. In this regard, the use of silicon (Si) supplementation offers a promising prospect since numerous studies have reported the beneficial role of Si in mitigating stresses imposed by biotic as well as abiotic factors including heavy metal stress. The fundamental mechanisms involved in the Si-mediated heavy metal stress tolerance include reduction of metal ions in soil substrate, co-precipitation of toxic metals, metal-transport related gene regulation, chelation, stimulation of antioxidants, compartmentation of metal ions, and structural alterations in plants. Exogenous application of Si has been well documented to increase heavy metal tolerance in numerous plant species. The beneficial effects of Si are particularly evident in plants able to accumulate high levels of Si. Consequently, to enhance metal tolerance in plants, the inherent genetic potential for Si uptake should be improved. In the present review, we have discussed the potential role and mechanisms involved in the Si-mediated alleviation of metal toxicity as well as different approaches for enhancing Si-derived benefits in crop plants.

## 1. Introduction

Plants, being sessile, are continuously exposed to many biotic and abiotic stresses affecting their growth and development. Among the abiotic factors affecting plants, heavy metal stresses have received increasing attention over the last several decades. The term heavy metal refers to any metallic element with relatively high density that is toxic even at low concentration. In general, heavy metals relate to a group of metals and metalloids with greater than 4 g·cm^−3^ atomic density [1]. Among the naturally occurring elements, 53 are categorized as heavy metals. The heavy metals include cadmium (Cd), nickel (Ni), lead (Pb), iron (Fe), zinc (Zn), cobalt (Co), arsenic (As), chromium (Cr), silver (Ag) and platinum (Pt), and the majority of them do not play an essential role in plants. Although naturally present in the soil, concentration of these heavy metals increases as a result of geologic and anthropogenic activities causing a harmful/toxic effect on both plants and animals [2]. Heavy metals retard plant growth by marginalizing the cellular functions of proteins, lipids, and elemental components of thylakoid membranes [3]. Moreover, heavy metals can be transported through the food chain into animals and humans, so their presence will cause a significant threat to human health [4]. Among means to reduce heavy metal toxicity in agricultural production, silicon (Si) is often reported for its potential to mitigate their adverse effects [3,5].

Silicon, a second most abundant element after oxygen in the earth crust, is considered as a quasi-essential element because of the numerous benefits it confers to plants, specifically under biotic and abiotic stress [6,7]. For instance, it is reported to alleviate a number of abiotic factors in plants including drought, salinity stress, lodging and heavy metal toxicity [8,9]. A possible role of Si in metal detoxification is attributed to alteration of plant cellular mechanisms and biochemical interactions with the external growth medium [10]. The positive effects of Si vary with the crop species and are usually more pronounced in plants that accumulate high concentrations of Si in their tissues [11,12]. The beneficial effects of Si are predominantly if not exclusively manifest when plants are subjected to stress [13]. Despite the abundant availability of Si in soils, the plant-available form is often limited in most soil types. Plant roots uptake Si in the form of silicic acid (H_4_SiO_4_) where the concentration ranges from 0.1 to 0.6 mmol·L^−1^ in the soil solution [14]. The Si concentrations in plants will vary according to the plant species, ranging from 1 to 100 g·kg^−1^, which represents the largest range of mineral elements [6]. This phenomenon is related to the different ability of plant species to uptake and transport Si, through a dedicated system of Si transporters [14,15]. In the present review, we discuss the role of Si in enhancing heavy metal tolerance in crop plants and the possible mechanisms by which Si achieves this feat.

## 2. Heavy Metal: From Essentiality to Toxicity

Heavy metals are classified into non-essential elements (Cd, Pd, Hg, Cr, As and Ag) being potentially toxic to plants and essential micronutrients (Cu, Zn, Fe, Mn, Mo, Ni, and Co) which are important for healthy growth and development of plants [16]. The essential heavy metals are involved in many important biochemical and physiological processes of plants. The principal functions of essential heavy metals include participation in the redox reaction of cellular processes and other molecular activities by being an integral part of several enzymes. In general, a plant grows normally as long as the supply of a given nutrient matches the plant’s requirement. Deficiency of nutrients will result in symptoms leading as far as mortality under extreme conditions. Typical deficiency symptoms arising from different metals in plants are presented in Supplementary Text 1. The presence of both essential and non-essential heavy metals in excess can lead to the reduction and inhibition of growth in plants, caused by biochemical, structural and physiological changes [17]. Higher concentrations of heavy metals also alter the uptake, accumulation, and translocation of the essential elements in plants [18]. Common toxic effects of heavy metals include inhibition of growth and photosynthesis, chlorosis, low biomass accumulation, altered nutrient assimilation, and water balance, and senescence, which ultimately can cause plant death [17].

## 3. Heavy Metal Toxicity and Crop Plants

**Figure 1:**
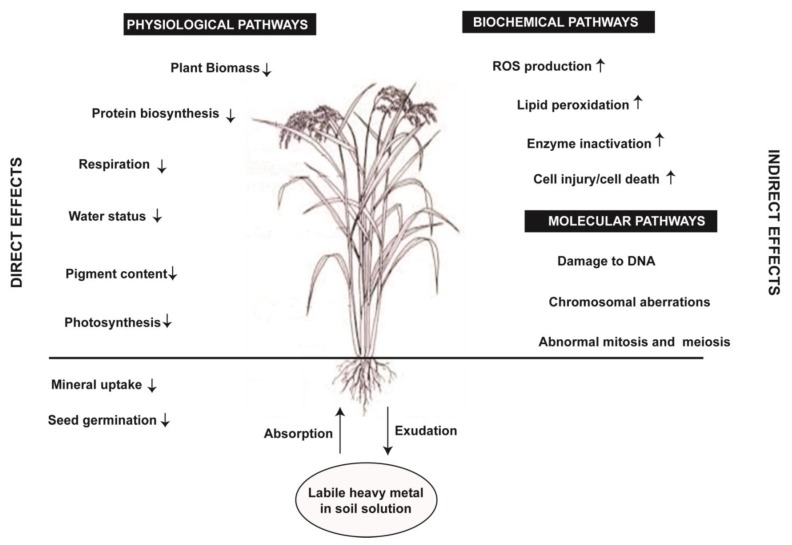
Heavy metal uptake by plant roots as well as their possible direct and indirect negative effects on crop productivity. The sign ↓ indicates decrease and the sign ↑ indicates increase.

Heavy metal contamination of agricultural soils has emerged as a critical and significant concern because of unfavorable ecological effects. The land used for crop production has been reported to be polluted with the excess of heavy metals, especially by Cd, Pb, and Zn in many parts of the world [19]. The agricultural land that has been polluted by the elevated concentration of heavy metals was shown to have highly adverse effects on plant metabolism and growth, soil biological activity, fertility, biodiversity, and health of humans and animals [18,19]. Several studies carried out to understand the toxic impact of heavy metals on plants highlighted many direct and indirect toxic effects of heavy metals in different crop species (Figure 1; Table 1; Supplementary Text 1). Several different approaches including the application of phyto-extracts, mobilizers and, more sustainably, the use of Si have contributed to the mitigation of heavy metal stress in plants [20,21].

## 4. Silicon: A Multifaceted Element for Alleviating Heavy Metal Toxicity in Crops

Silicon derived enhancement in plant tolerance to heavy metal toxicity is well documented, and the beneficial role of Si in detoxification can be ascribed to both external (growth media) and internal plant mechanisms [21,22]. The external mechanism of elevating heavy metal tolerance is mainly due to the increased pH by silicate application resulting in metal silicate precipitates that decrease the metal phyto-availability [22]. In plants, Si affects the translocation and distribution of metals in various plant parts and allows them to survive under higher metal stress [23]. Given that plants vary in their ability to accumulate Si, higher accumulators such as monocots will usually obtain greater benefits, even though metal toxicity in both monocots and dicots can be alleviated by Si [24,25]. In rice, Si-rich amendments showed a reduction of heavy metal accumulation, as well as increased growth in multi-metal (Cd, Zn, Cu, and Pb), contaminated acidic soil [26]. Silicon is also reported to increase seedling biomass and decrease Zn concentration in both roots and shoots of rice seedlings, and the xylem sap flow. In addition, Zn accumulation was significantly suppressed by Si supply in different plant parts such as roots and leaves of cotton and maize [27,28]. Shi et al. [29] reported that Si minimizes Cd metal toxicity by reducing ion absorption and translocation from root to shoot in rice seedlings. Silicon application was also found to reduce lipid peroxidation and fatty acid desaturation in plant tissues and improve the growth and biomass of plants under heavy metal stress [4].

Silicon can also be effective in alleviating Al toxicity in barley plants [30]. Similarly, decreased Al content with Si application was observed in the stem, roots, and leaves of peanut and rice seedlings [31,32]. Silicon amendments as an *alternative* detoxification method for Al toxicity have been reported in sorghum, tomato, soybean and maize [21]. Considerable reduction of Si pools in agricultural soils results from regular removal of Si-rich litter during the crop harvest [33]. Hence, external application of Si in agriculture will become a trend in the near future to compensate its depletion in soils, simultaneously reaping its benefits of improving plant growth and alleviating heavy metal toxicity.

## 5. Silicon-Mediated Mechanisms of Metal Detoxification

**Figure 2:**
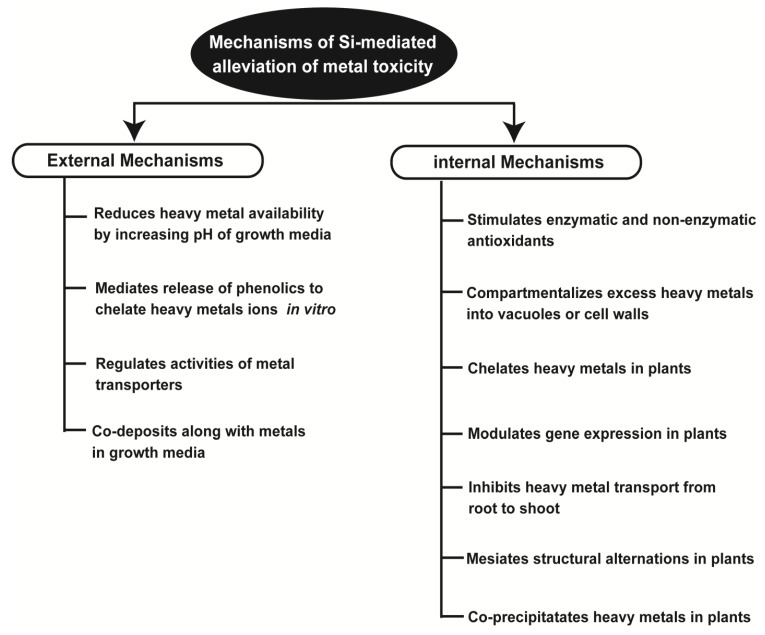
Different external and internal mechanisms used by silicon to mitigate the toxic effects of heavy metals.

Several mechanisms explaining Si-derived benefits towards metal detoxification have been proposed. The widely accepted mechanisms include toxic metal immobilization in the soil (at soil level), and stimulation of enzymatic and non-enzymatic antioxidants, co-precipitation of metals, metal ions chelation, compartmentation, and structural alterations of plant tissues and alteration in molecular responses (at plant level) (Figure 2; Table 2).

### 5.1. Silicon Mediated Immobilization of Toxic Metal in the Soil

Immobilization of toxic metals is the much simpler mechanism to explain Si-derived benefits. The toxic metal immobilization in the soil through Si application has been reported in several studies [21]. The immobilization took place either due to the increased soil pH or changing metal speciation in soil solution through the formation of silicate complexes. In rice, application of Si-rich amendments (fly ash and steel slag) was found to increase soil pH from 4.0 to 5.0–6.4, and decreased the phyto-availability of heavy metals by at least 60%, which further suppressed metal uptake [26]. Similarly, in banana, reduced uptake of Pb has been reported with Si application in Pb contaminated soil [34]. The decreased bio-availability of Pb in banana was found to be associated with significantly increased soil pH and decreased proportion of exchangeable Pb in soil [34].

In addition, Si helps by changing the speciation of metals from toxic to nontoxic form by the formation of silicate complexes in the soil solution. In Si-amended soil, mostly Cd was found in the form of oxides or adsorbed by Fe-Mn oxides [35]. It has been proposed that the bio-availability of Al to plants in the presence of Si can be limited by forming Al-Si complexes like hydroxyaluminosilicate (HAS) [36]. In aqueous solution, soluble silicate hydrolyzes and produces gelatinous metasilicic acid (H_2_SiO_3_) which can absorb heavy metals, or lead to deposition of heavy metals into their silicates in Si-rich soil [26], both of which reduce the metal concentration available for uptake. Zhang et al. [37] have shown that the Si application in Cr-contaminated soil can markedly decrease the amount of exchangeable Cr by accelerating the precipitation of organic matter bound Cr fraction. Similarly, Shim et al. [38] have observed the reduced metal mobility in Pb contaminated soil by the Si application. Subsequent investigation with X-ray diffraction analysis revealed the formation of insoluble Pb-silicate in the soil. Similarly, effects have been observed in the case of Cd and Zn contaminated soil where Si application accelerated the formation of more stable fractions of Cd and Zn [28].

### 5.2. Stimulation of Antioxidant Defense System

Heavy metal stress induces an excess formation of reactive oxygen species (ROS), which results in several metabolic disorders in crop plants [39,40]. In this context, the enzymatic and non-enzymatic antioxidant system stimulated by Si helps to lower oxidative stress by reducing the production of ROS. Alleviation of Mn toxicity by Si in cucumber was attributed to a significant reduction in lipid peroxidation (LPO) intensity caused by excess Mn, and to a significant increase in enzymatic (*Superoxide dismutase*, *ascorbate peroxidase*, and *glutathione reductase*), and non-enzymatic (ascorbate and glutathione) antioxidants [41]. Similarly, under Cd stress, Si application reduced the H_2_O_2_ and electrolytic leakage (EL) in *Solanum nigrum* [42]. Decreased contents of an oxidative stress related compound like malondialdehyde (MDA), H_2_O_2_ and EL were observed with Si application in plants under Cd [43], Zn [27], and Pb stress [44]. Thiobarbituric acid reactive substances (TBARS), a widely used marker for reactive oxygen contents, were found to be reduced significantly with Si supplementation in rice and maize plants grown under Cd stress condition [39]. Similarly, under Cd stress, the effect of Si supplementation on antioxidants has also been observed in many crop plants including maize [45], wheat [43], rice [23,46] and peanut [47]. Enhanced antioxidant enzyme activities as well as activities of non-enzymatic antioxidants like glutathione, non-protein thiols, and ascorbic acid have been reported in several plant species under heavy metal stress [48,49]. Si-mediated detoxification through stimulating enzymatic and non-enzymatic antioxidants has also been observed under Pb, Mn, Zn and Cu stress [20]. In light of the previous studies, application of Si appears to induce the antioxidant system in plants, thereby improving stress resistance, but it is still unclear if this results from a direct or indirect action of Si [50].

### 5.3. Compartmentation within Plants

Enhanced compartmentation of metal elements in plant tissues has been observed with Si supplementation in several studies. Williams and Vlamis [51] observed for the first time that the effect of Si in alleviating Mn toxicity in barley was not the result of a reduction in Mn concentration, but rather of improved compartmentation within the leaf tissues. Another level of compartmentation, mostly regulated through the translocation activity leading to the increased metal concentration in plant roots compared to shoots, has been widely reported with Si supplementation [52,53]. The Si treatment was observed to reduce transport of Zn from roots to shoots and increase the binding of Zn to the cell wall, thus decreasing Zn concentration in the rice shoots [53]. In wheat, Si application found to reduce Cd translocation from root to shoots and grains [54]. In rice, Shi et al. [29] observed a Si-decreased root to shoot translocation of Cd by 33%. Microscopic analysis performed by Shi et al. [29] clearly showed deposition of Cd near the endodermis and epidermis; however, a high amount of Si was found to be deposited near the endodermis than in the epidermis. The Si deposition at endodermis seems to play a crucial role in decreasing Cd translocation from the epidermis to the endodermis. In addition, with Si application in rice, decreased Cd accumulation in shoots was found to be associated with the increased compartmentalization of Cd in the root cell walls [23]. Considerable reduction in the symplasmic concentration of Cd and increase in the apoplastic concentration in maize grown with Si supplementation have been reported by Vaculik et al. [55]. Silicon was also observed to increase the Mn localization in the cell wall in cucumber [56] and cowpea [57] under Mn stress and in rice, under Zn stress [26]. Furthermore, in Si-treated plants, less Mn was located in the symplast (<10%) and more Mn was bound to the cell wall (>90%) compared to control plants (about 50% in each compartment) [58]. Hence, Si-mediated detoxification of heavy metals through their compartmentation into different plant tissues might be a key mechanism supporting the beneficial role of Si.

### 5.4. Chelation Mediated Metal Toxicity Reduction with Silicon Application

The Si-mediated heavy metal detoxification predominantly includes the chelation of metal by flavonoid-phenolics or organic acids. Increased phenol exudation up to 15 times has been observed in maize upon Si supplementation. The phenolic compounds like catechin and quercetin have high Al-chelating activity, which can alleviate Al toxicity in the root tip apoplast [10,59]. Barceló et al. [60] revealed a considerable increase of malic acid concentration upon Si application in maize under Al stress. The reduced Al toxicity was found to be correlated with the chelating of Al with malic acid. In bamboo, Si has also been observed to increase the concentration of Cu(I) S-ligands that chelate Cu as well as increase the Cu sequestration in a less toxic form [61]. Keller et al. [52] have observed reduction in translocation of Cu from root to shoot in wheat grown with Si supplementation. The altered translocation of Cu may be because of the increased proportion of citrate, malate, and aconitate in roots of wheat seedlings. Taken together, these studies suggest that Si may indirectly promote the chelation of heavy metals in plants, thereby reducing their phytotoxicity.

Schaller et al. [62] recently reported that Si availability was significantly correlated to phosphorus (P) mobilization in Arctic soils. The results confirmed that the addition of Si significantly increases P mobilization by mobilizing Fe(II)-P phases from mineral surfaces as well as increases soil respiration in P deficient soils. The study highlights the Si as an important component regulating P mobilization in Arctic soils. Besides an important nutrient element, P supplementation also plays an important role in biochemical activities in the soil. In this regard, significant efforts have been performed to understand the effects of P mobilization on bioavailability of heavy metals [63]. A considerably high number of studies have shown the importance of P compounds to reduce bioavailability of heavy metals by immobilizing the metal ions in the soil [64]. Therefore, Si mediated P mobilization seems a valuable option for the sustainable management of P availability as well as for the minimizing losses that occurred due to heavy metals in agro-ecosystems.

### 5.5. Regulation of Gene Expression

Phytochelatin synthesis represents one of the major heavy metal detoxification mechanisms in plants [65]. The Si-mediated mitigation of metal toxicity is also attributed to its role in altering gene expression. In Arabidopsis grown under Cu stress, Si supplementation was observed to stimulate the genes governing the production of metallothioneins (MTs), a well-known chelating agent [66]. Similarly, significantly downregulated expression of the genes encoding heavy metal transporters (*OsHMA2* and *OsHMA3*), and upregulation of the genes responsible for Si transport (*OsLSi1* and *OsLSi2*) has been observed with Si supplementation in rice [3]. Similarly, the enhanced expression of *phytochelatin synthase* 1 (*PCS1*) and decreased expression of the *metallothionein* gene (*MT1a*) was associated with Si supplementation in Arabidopsis under Cu stress [67]. Recently, Ma et al. [68] have shown that Si supply under Cd stress in rice upregulated the expression of *OsLsi1* (encoding for Si transport NIP-III (Nodulin 26-like intrinsic proteins-III) Aquaporin) and downregulated the expression of *Nramp5*, a gene involved in the Cd transport. However, many plant species particularly belonging to family *brassicaceae* [69], *solanaceae* [70], and *Linaceae* [71] does not carry any Si-transporter (NIP-III Aquaporin) and are known as poor Si accumulators. However, several reports suggesting Si derived benefits in such species make it more difficult to understand the molecular consequences [72,73]. So far, the Si-mediated mechanisms for the reduction of metal toxicity are less understood at the genetic and molecular levels. A better understanding of the gene expression dynamics involved in Si-driven alleviation of metal toxicity is necessary to properly decipher the molecular mechanism underlying this phenomenon. Still, limited efforts have been directed toward explaining Si-mediated transcriptomic changes in plants, and none of those were related to metal toxicity.

### 5.6. Structural Alterations Related to Metal Stress Tolerance in Plants

Morphological and anatomical features of crop plants get improved with Si supplementation that helps to overcome the adverse effects of heavy metal stress. Notable examples where increased plant height, root length, number of leaves and leaf size have been observed with Si application to plants under Cd, Zn and Pb stress [74,75]. Ali et al. [76] observed that Si + Cr treatments increased plant height, the number of tillers, root length, and leaf size of barley plants compared to plants treated with Cr only. Similarly, root length and shoot size were significantly increased with Si compared to without Si treatment [74]. Doncheva et al. [77] reported that the Si application increased the leaf-epidermal-layer thickness in maize plant under Mn stress. Silicon was also reported to increase xylem diameter, epidermis, mesophyll and the transverse area of collenchymas and mid-vein under Cd and Zn stress [28,78]. The accelerated development of the Casparian bands, suberin lamellae, and root vascular tissues was observed in maize with Cd + Si treatments [45,55]. Similarly, higher growth of suberin lamellae in the endodermis particularly near the root tips has been observed with Si application in rapeseed and Indian mustard grown under Cd stress [79]. In wheat, Greger et al. [80] reported the formation of apoplastic barriers in the endodermis closer to the root apex in the presence of Si in Cd-treated plants. Thus, structural alterations induced by Si under metal stress may explain the alleviation of heavy metal toxicity.

### 5.7. Co-Precipitation of Metals by Silicon Application in Soil

Many studies suggest that the co-precipitation of metals by Si leads to the alleviation of heavy metal stress in plants. For example, Si treatment in plants under Al stress was suggested to form aluminosilicates or hydroxyaluminosilicates (or both) in the apoplast of the plant root apex leading to Al detoxification [22,60]. In *Minuartia verna* (Si-accumulating dicot), Si was reported to co-precipitate Zn as their silicates in the leaf epidermis cell wall [81]. Gu et al. [26] observed the co-precipitation of Si with Cd in the stem of rice that lowered heavy metal concentration in leaves. Similarly, Si was proposed to form a complex with Zn at the leaf surface of *Cardaminopsis halleri* [82]. Zhang et al. [23] observed synchronous accumulation of Si and Cd in the middle and border of phytoliths in rice shoots. However, there are some contrasting reports, such as ones by Rizwan et al. [83] and Keller et al. [52], where Cu and Cd were not found in the leaf phytoliths of wheat. These reports suggest the possibility of a mechanism other than the co-precipitation involved in Cu detoxification in wheat plants. Similarly, Dresler et al. [84] report also support the notion since Si–Cd complexes were not observed in maize plants treated with Si under Cd stress.

## 6. Approaches for Improving Silicon Accumulation in Crop Plants

**Figure 3:**
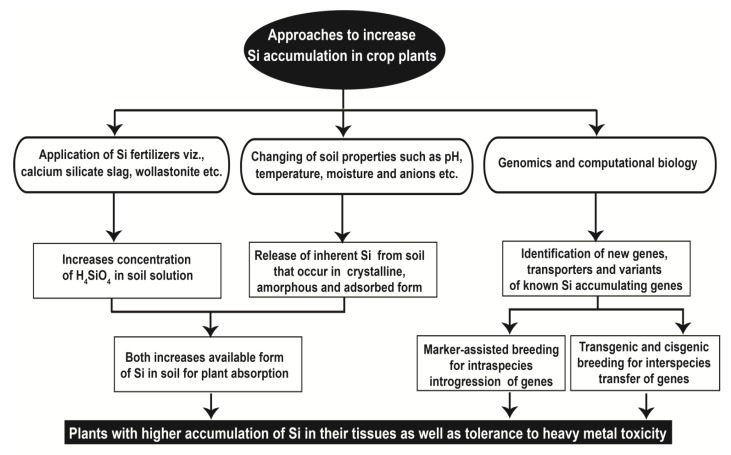
The different approaches that can be utilized for improving silicon accumulation to enhance metal stress tolerance in crop plants.

The beneficial effect of Si for mitigating the toxic effects of heavy metals is usually apparent in plants that accumulate high levels of Si in their shoots [53,85]. Silicon accumulation in plants depends upon the availability of silicic acid (H_4_SiO_4_) in the soil as well as the inherent capacity of the plant for Si uptake. Silicon availability in a soil can be improved by applying Si-rich fertilizers or by modifying soil properties, whereas genetic modification or selection can improve the inherent capacity a species to accumulate Si. The different approaches used for increasing Si accumulation in crop plants are presented in Figure 3 and are discussed in the following sections.

### 6.1. Silicon Fertilization

The estimated amount of Si removed annually by different crops on a global scale is between 210 and 224 million tons [86]. Continuous and intensive cropping of Si-accumulator crops results in a significant reduction of plant-available Si in the soil [87]. Desplanques et al. [88] noted that five years of continuous cultivation of rice could exhaust most of the available Si from the soil. In addition, certain soils contain low levels of Si, particularly the plant-available form, and these soils include Oxisols, Ultisols, and Histosols as well as soils that are composed of a large fraction of quartz [89]. Silicon is absorbed by plants in the form of monosilicic acid (H_4_SiO_4_) and its concentration in the soil solution will determine the amount a plant accumulates [90]. Fertilization can rapidly increase the concentration of H_4_SiO_4_ in the soil and has become a standard practice in areas with intensive cropping systems, particularly for those soils that are inherently low in soluble silicon [91,92]. Several studies have shown a beneficial effect of an adequate supply of Si in growth medium not only for high Si-accumulating plants such as rice [46] and sugarcane but also for poor accumulators like tomato, cucumber, strawberry and orange [93,94] (Table 2). On the other hand, some studies have also shown that the fertilization regime can influence the amount of Si that will be available to the plant [94]. With respect to the alleviation of heavy metal tolerance, Ning et al. [95] and Jarosz et al. [93] observed less concentration of Zn and Cu in fruits of plants grown with Si-based fertilizer. However, most of the studies to date have been carried out in greenhouses, and there is a lack of data supporting the performance of Si-based fertilizers in large-scale field experiments.

### 6.2. Modification of Soil Properties

The abundance of Si in soils does not necessarily relate to the concentration of plant-available Si [91]. The concentration of H_4_SiO_4_ in the soil solution is influenced by many soil factors such as pH, temperature, weathering of soil, moisture, redox potential, amounts of clay, mineral, organic matter and Fe/Al oxides/hydroxides [86]. The solubility of both the crystalline and the amorphous silica is approximately constant between pH values 2 and 8.5 and increases rapidly at pH ~9. The soil pH also affects the formation of Si-complexes with other elements, for instance, the amount of monosilicic acids that is absorbed by Fe/Al oxides increases from pH 4 to pH 10. Kaczorek and Sommer [96] have revealed that, under the conditions of soil acidification, the number of free silica increases. Similarly, Höhn et al. [97] reported an increase of Si available in the soil with a decrease in pH. In this regard, the application of acid-producing fertilizer increases the concentration of H_4_SiO_4_ in the soil solution, whereas liming and high organic matter content results in a reduction in the concentration and mobility of the H_4_SiO_4_.

### 6.3. Approaches for Genetic Gain

Identification of two Si-transporters genes using low silicon (*Lsi*) rice mutants by Ma et al. [98,99] are the milestone discoveries that accelerated Si research many folds. One of the genes is a passive influx transporter (*OsLsi1*) belonging to an NIP group of the aquaporin family, which is responsible for the uptake of Si from soil into the root cells [15,100]. Several homologs of the *Lsi1* have been identified and functionally validated in different plant species (Table 3, Supplementary Text 1: Table S1). Another gene, *Lsi2*, encodes an active efflux transporter and belongs to the cation transporter family (Table 3; Figure S1). The information about the *Lsi1* and *Lsi2* was helpful to understand the uptake of Si from the root and subsequent trans-location from roots to aerial parts of the plant [101]. It also provided an opportunity to explore transgenic approaches for the enhancement of Si-uptake, particularly in poor accumulator plant species. In Arabidopsis, a well-known poor accumulator, heterologous expression of wheat Si-transporter (*TaLsi1*) showed increased Si absorption by several folds [102]. Similarly, functionally characterized Si-transporter (mostly with *Xenopus oocyte* assays) from different species could be utilized efficiently for the development of transgenic crop plants (Table S1). Recently, Deshmukh et al. [103] showed categorization of plant species as poor or high Si-accumulator based on the presence of *Lsi1* homolog. The interspecies variation can be sufficiently explained by the characterization of *Lsi1* homolog. Considerable interspecies variation for Si concentration has been reported, ranging from 0.1 to 10%, although these variations appear to be more limited at the intraspecies level [14]. For example, in sugarcane (*Saccharum officinarum*), the Si concentration in the shoots of different genotypes has been observed to range from 6.4 to 10.2 mg^−1^ [104]. Similarly, in a survey of about 400 cultivars of barley (*Hordeum vulgare*), the Si concentration in barley grain revealed a variation, ranging from 1.24 to 3.80 mg·g^−1^ in barley grains [105]. In rice, japonica rice cultivars usually accumulate more Si than indica rice cultivars [106]. Quantitative trait loci (QTLs) governing the intra-species variation have been identified in rice. However, these QTLs do not collocate with the major genes *Lsi1* and *Lsi2*, and the discovery of *Lsi1* and *Lsi2* enhanced molecular understanding of Si-uptake has limited use for crop improvement. The gene(s) responsible for the genotypic differences have yet to be identified. Efforts towards the identification of such genes will help to develop cultivars with better Si uptake through breeding approaches.

## 7. Conclusions

Natural and anthropogenic activities have resulted in a higher concentration of toxic metals in the agricultural farmlands leading to the severe adverse effects on crop production and productivity as well as human health. In this regard, Si has emerged as a practical option to reduce phytotoxicity and accumulation of toxic metal in plants. Beneficial effects derived from Si have been reported in several studies and have been explained through different possible mechanisms, although more experiments are needed to validate them. Many studies argue that the use of Si-based fertilizers constitutes a promising approach to alleviate heavy metal toxicity and large-scale field studies should be undertaken to define with precision the extent of Si-derived benefits. At the same time, the enhancement of the genetic potential of plants to uptake Si would represent an interesting avenue to optimize their responses to Si. The recent advances in the field of genomics, computational biology, and high-throughput phenotyping will facilitate the deciphering of the genetic basis of Si accumulation in crop plants, which in turn should accelerate the development of varieties accumulating higher levels of Si. This strategy would fit very well in a program of sustainable agriculture to improve heavy metal tolerance in plants.

## Figures and Tables

**Table 1:** Phytotoxicity of heavy metals on growth, development, and metabolism of different crop species.

Crop Species	Heavy Metal	Phytotoxicity	References ^#^
Rice	Hg	Reduction in grain yield; reduced tiller and panicle formation; decrease in plant height	S1
	As	Reduction in seed germination and seedling height; reduced leaf area and dry matter production	S2
	Cd	Inhibition of root growth; The appearance of black spots in the cortex and pericycle	S3
Wheat	Pb	Reduced shoot/root length, shoot fresh/dry weights, number of tillers; decreased photosynthetic pigments such as chla and chlb	S4
	Pb	The decrease in the contents of Chl*a*, Chl*b* and proline content; growth inhibition and a decrease in dry weights of plant parts	S5
	Ni	Reduction in the total shoot and root lengths; decrease in Chl*a* and Chl*b* contents and enhancement of chlorophyll a/b ratio	S6
	Cd	Reduction in seed germination; decrease in plant nutrient content; reduced shoot and root length	S7
	Cr	Inhibition of shoot and root growth	S8
	Ni	Reduction in plant nutrient acquisition	S9
Maize	Cd & Zn	Reduction in shoot/root biomass; decrease in length as well as dry weights of shoot/root	S10
	Cd & Ni	Inhibition of root growth; reduction in the length of primary roots	S11
	Cd	Inhibition of shoot and root growth	S12
Barley	Cd	Reduction of Chl*a*, Chl*b*, and carotenoids; decreased plant growth and biomass	S13
	Cu & Cd	Reduction of plant dry weights, root length, and shoot height; alteration of photosynthetic pigments and lipid peroxidation	S14
	Cd & Zn	Reduction of total biomass; decrease in the length of roots and leaves	S15
Sorghum	Cu & Cd	The decrease in root diameter, width, and thickness of leaf midrib and diameter of xylem vessels; reduction of yield and yield contributing traits	S16
	Cd	The decrease in chlorophyll pigments, plant growth, and root characteristics	S17
Oat	Pd	Inhibition of enzyme activity which affected CO_2_ fixation	S18
Soybean	Hg	Reduction of oil content; changes in major and minor fatty acid concentration of soybean seed	S19
	Cd	The decrease in the accumulated amount of dry matter as well as the content of mineral elements; inhibition of chlorophyll biosynthesis; decrease in the Mg uptake	S20
	Co & Pb	Reduced seed germination, seedling growth, leaf area, root development, and biomass production	S21
	Cd	Reduction of nodulation and leghaemoglobin content; a decrease in crop productivity	S22
Bean	Cu	Root malformation and accumulation of Cu in plant roots; reduction of root growth	S23
	Zn	The decrease in plant growth, development, and metabolism; induction of oxidative damage	S24
	Zn	Reduction of photosynthetic pigments including Chl*a* and Chl*b*; disruption of absorption and translocation of Fe and Mg into the chloroplast	S25
Chickpea	Cd	Inhibition of seed germination and root growth; disruption of vascular tissues as well as associated tissues	S26
	Cu	Reduction of root/shoot length and RWC; lipid peroxidation	S27
	Co	Inhibition of photosynthetic process and activity of antioxidative enzymes; The increase in proline content and lipid peroxidation	S28
Pigeonpea	Ni	The decrease in stomatal conductance and chlorophyll content; decreased enzyme activity which affected the Calvin cycle and CO_2_ fixation	S29
	Pd & Cd	Reduction in photosynthetic activity; decrease in chlorophyll content and stomatal conductance	S30
	Hg & Cd	Reduction of germination percentage, root and shoot length, fresh and dry weight of seedlings; inhibition of root elongation	S31
Faba bean	Mn	Mn accumulation in shoot and root; reduction of the shoot and root length; chlorosis	S32
	Cd	Cd accumulation in shoot and root; decrease in photosynthetic pigments as well as root fresh and dry weights	S33
Pea	Mn	The decrease in Chl*a* and Chl*b* content; reduction in relative growth rate; reduced photosynthetic O_2_ evolution activity and photosystem II activity	S34
	Zn	Reduction in chlorophyll content; alteration in the structure of chloroplast; reduction in photosystem II activity; reduced plant growth	S35
Mung bean	Co	Reduction in antioxidant enzyme activities; decrease in plant sugar, starch, amino acids, and protein content	S36
Groundnut	Cd, Pb & As	Reduction in germination rate, root length, shoot length, biomass and seedling vigor index	S37
	Cr	Increase in lipid peroxidation; decrease in photosynthetic pigments; induced oxidative stress	S38
	Ni	Reduction of seed germination, root/shoot growth and fresh weight of seedlings	S39
Cotton	Cd	The decrease in plant height, biomass and leaf area; reduction of Chl*a*, Chl*b*, photosynthetic rate, stomatal conductance, and transpiration rate	S40
	Pb	The decrease in net photosynthetic rate, stomatal conductance, transpiration rate, water use efficiency, chlorophyll, carotenoids, and the Soil Plant Analysis Development (SPAD) chlorophyll meter value	S41
Tomato	As	The decrease in leaf fresh weight; Reduced fruit yield	S42
	Co	Reduction in plant nutrient content	S43
	Cr	Decrease in plant nutrient acquisition	S44
	Hg	Reduction in germination percentage; reduced plant height; reduction in flowering and fruit weight; chlorosis	S45
Canola	As	Wilting, chlorosis and stunted growth	S46
*Brassica juncea*	Cd & Pb	Reduction of growth and biomass yield; decrease of chlorophyll and carotenoid pigments	S47
Garlic	Cd	Reduced shoot growth; Cd accumulation	S48
Onion	Cr	Inhibition of germination process; reduction of plant biomass	S49
Radish	Co	Decrease in shoot and root length as well as total leaf area; reduction in chlorophyll content, plant nutrient content and antioxidant enzyme activity	S50

^#^ Detailed list of references are provided in the Supplementary Text 2.

**Table 2:** External and internal silicon-mediated mechanisms for enhancing tolerance of plant’s against heavy metal toxicity.

Crop	Heavy Metal	Mechanisms	References ^#^
Rice	Cd, Zn, Cu & Pb	Immobilization of heavy metals in culture media and decrease of phytoavailability which further suppressed metal uptake	S51
	As	Overcomes heavy metal uptake by competes with arsenate ions for root entry point	S52
	Zn	Strong binding of Zn in the cell wall of less bioactive tissues, especially in sclerenchyma of root	S51
	Pb	Preventing Pb transfer from rice roots to aboveground parts, and blocking Pb accumulation in rice grains	S53
	Cd	Si bound to cell wall inhibits apoplastic Cd uptake by covalently bonding with Cd and trapping Cd as it diffuses through the cell wall and intracellular spaces.	S54
	Cd	Increased the activities of antioxidant enzymes and preventing membrane oxidative damage of plant tissue	S55
	Cd	Decreased Cd accumulation in rice shoots by compartmentalization of Cd in the root cell walls	S56
Maize	Cd & Zn	Increased diameter of xylem, thickness of leaf mesophyll and epidermis, and transversal area occupied by collenchyma and mid vein	S57
	Al	Formation of hydroxyaluminosilicates in the apoplast of the root apex reducing the mobility of apoplastic Al	S58
	Cd	Formation of colloidal silicon in cell walls which has high specific adsorption property to Cd preventing Cd uptake into the cell	S59
	Cd	Cd co-precipitation with silicates, resulting in strong binding of Cd to cell walls, thereby reducing the concentration of Cd in the symplast	S60
	Zn	Formation of less soluble zinc-silicates in the cytoplasm	S61
Wheat	Cd	Enhanced antioxidant enzymes activities, and preventing lipid peroxidation as well as membrane oxidative damage of plant tissue	S62
	Cd	Decreased Cd uptake as well as translocation of Cd to shoots and grains	S63
	Cu	Cu form complex with organic acids and reduced the Cu translocation to shoots	S64
	Cd	Formation of apoplasmic barriers in endodermis closer to the wheat root apex	S65
Barley	Al	Exclusion of Al from the subtending tissue as a result of silicon deposition at the epidermis, restricting total overall Al uptake into the root	S66
	Cr	Increased plant height, number of tillers, root length and leaf size of barley plants	S67
Cucumber	Mn	Reduction of lipid peroxidation, and increase of enzymatic and non-enzymatic antioxidants levels	S68
	Mn	Si increased Mn fraction in the cell wall of shoots, thereby reduced Mn concentration of symplast	S69
Cowpea	Mn	Si reduced the apoplastic Mn concentration and modify the cation binding capacity of the cell wall	S70
	Mn	Enhanced adsorption of Mn on cell walls reducing the amount of soluble apoplastic Mn	S71
	Mn	Co-precipitation of Si and Mn in leaf apoplast of cowpea plants, and increases Mn fraction in the cell wall of shoots	S71
Peanut	Cd	Increased activities of antioxidant enzymes; inhibition of Cd transport from roots to shoots	S72
Arabidopsis	Cu	Stimulated the genes responsible for the production of metallothioneins (MTs) that can chelate toxic metals	S73
Rapeseed	Cd	Mediated extensive development of suberin lamellae in endoderm closer to the root tips	S74
*Minuartia verna*	Zn	Co-precipitation as Zn silicates in the cell walls of leaf epidermis	S75
*Cardaminopsis halleri*	Zn	Formation of Si–Zn complexes in leaves surface of Cardaminopsishalleri grown on a Zn contaminated soil amended with Si.	S76
Pakchoi	Cd	Increased activities of enzymatic and non-enzymatic antioxidants levels, protein thiols (NPT) and ascorbic acid	S77
Cotton	Cd	Enhanced activities of antioxidant enzymes as well as reduced electrolytic leakage, malondialdehyde and hydrogen peroxide contents, thereby preventing plant tissue from oxidative damage	S78
	Pb	Increased the activities of antioxidant enzymes and preventing membrane oxidative damage of plant tissue	S41

^#^ Detailed list of references are provided in the Supplementary Text 2.

**Table 3:** Influx/efflux Si transporters as well as their ortholog’s identified in different crop species.

Crop Species	Transporter	Type	Expression Site	References ^#^
Maize	ZmLsi1	Influx	Root	S79
	ZmLsi6	Influx	Leaf Sheaths and blades	S80
	ZmLsi2	Efflux	Roots	S80
Rice	OsLsi1	Influx	Roots	S81
	OsLsi2	Efflux	Roots	S82
	OsLsi6	Influx	Leaf	S83
	OsLsi3	Influx	Panicles	S84
Barley	HvLsi2	Efflux	Root	S80
	HvLsi1	Influx	Root	S85
	HvLsi6	Influx	Leaf Sheaths and blades	S86
Soybean	GmNIP2-1	Influx	Root and shoot	S87
	GmNIP2-2	Influx	Root and shoot	S87
Wheat	TaLsi1	Influx	Root	S88
Pumpkin	CmLsi1	Influx	Root and shoot	S89
	CmLsi2	Efflux	Root and shoot	S89
Horsetail	EaNIP3;1	Influx	Root and shoot	S90
	EaNIP3;3,	Influx	Root and shoot	S90
	EaLsi2-1	Efflux	Root and shoot	S91
	EaLsi2-2	Efflux	Root and shoot	S91
Potato	StLsi1	Influx	Root and leaves	S92
Tomato	SlNIP2-1 (V140del) *	Influx	Root and leaves	S93

* mutated version where removal of Valine at potion 140 in non-functional wildtype turn it into a functional Si-transporter; ^#^ Detailed list of references are provided in the Supplementary Text 2.

## Supplementary Materials

The following are available online at https://www.mdpi.com/2223-7747/8/3/71/s1, **Figure S1:** Diagram showing the absorption of Si in rice plants. Silicon enters the exodermis in the form of silicic acid through specific influx transporter (Lsi1) and leaves into the cortex through active transporters (Lsi2). In the cortex, silicic acid moves apoplastically until it reaches the endodermis, where the silicic acid is loaded into the stele by Lsi1 and Lsi2 transporters. The solid green line shows the path of Si transport. **Table S1:** Details of silicon transporter genes from different plant species were validated using oocyte assay or transgenic approaches. **Supplementary Text 1:** Detailed discussion about the deficiency symptoms caused by different metals in plants, toxic effects of heavy metals on growth, development, and metabolism of different crop species. **Supplementary Text 2**: Details of references provided in Table 1, Table 2 and Table 3.
