# Supplementary material for: Role of Silicon in Mitigation of Heavy Metal Stresses in Crop Plants
Source: Plants (Basel). 2019 Mar 21;8(3):71. doi: 10.3390/plants8030071 (PMC6473438; doi:10.3390/plants8030071)
Supplement: Supplementary file 1 [file plants-08-00071-s001.zip › Figure S1.docx]

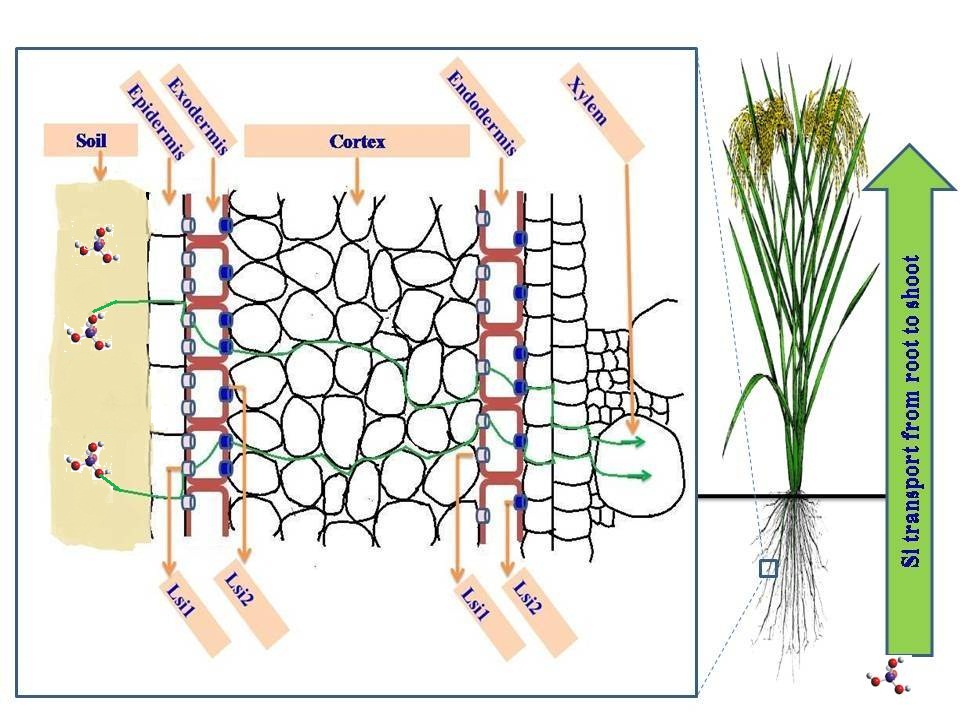


**Silicic**

**acid**

**Figure S1.** Diagram showing absorption of Si in rice plants. Silicon enters the exodermis in the form of silicic acid through specific influx transporter (*Lsi1*) and leaves into the cortex through active transporters (*Lsi2*). In the cortex silicic acid moves apoplastically till it reaches the endodermis where the silicic acid is loaded into the stele by Lsi1 and Lsi2 transporters. The solid green line shows the path of Si transport.
