# Supplementary material for: Role of Silicon in Mitigation of Heavy Metal Stresses in Crop Plants
Source: Plants (Basel). 2019 Mar 21;8(3):71. doi: 10.3390/plants8030071 (PMC6473438; doi:10.3390/plants8030071)
Supplement: Supplementary file 1 [file plants-08-00071-s001.zip › Supplementary Text1.docx]

Supplementary Text 1

Deficiency symptoms caused by different metals in plants

Zinc is a cofactor for more than 300 enzymes and 200 transcription factors associated with the maintenance of membrane integrity, auxin metabolism, and reproduction [1]. In addition, Zn is required to maintain the integrity of ribosomes, and also takes part in the formation of carbohydrates and catalyzes the oxidation processes in plants. The deficiency of Zn in plants affects directly leaf and stem expansion as well as plant development, and is also reported to have consequences on chlorophyll levels and possibly necrosis [2]. Zn deficiency is also associated with an impairment of carbohydrate metabolism and protein synthesis [3]. Cu is a constituent of the primary electron donor in photosystem I of plants and is a cofactor of oxidase, mono-oxygenase and di-oxygenase as well as of enzymes involved in the elimination of superoxide radicals (e.g., superoxide dismutase and ascorbate oxidase) [4]. Moreover, it takes part in carbohydrate and protein metabolism, lignin formation, and perhaps is required for symbiotic N fixation. Ni is required by plants for proper seed germination and is also a metal component for enzyme urease that catalyzes the conversion of urea to ammonium [5]. Previous studies have revealed Ni to be beneficial for N metabolism in legumes and other plants in which ureides are important in metabolism [6]. Mn is required for photosynthetic reactions involving the enzymes such as mallic dehydrogenase and oxalo-succinic decarboxylase, and is also important for water splitting at photosystem II and for superoxide dismutase. In addition, Mn is involved in N metabolism and CO2 assimilation and chloroplasts are the most sensitive of cell organelles to Mn deficiency [7]. Grey speck in oats and marsh spot in peas are two well known Mn deficiencies in arable crops. Furthermore, white streak in wheat and an interveinal brown spot in barley are also symptoms of Mn deficiency [8]. Cobalt (Co) is essential for growth of the rhizobium bacteria involved in legume nodulation and fixation of atmospheric nitrogen in legumes. Although Co has not been essential for higher plant growth, but modulating bacteria need it for fixing atmospheric N in legumes, and its deficiency results reduced Vitamin B12 production and lower nitrogen fixation. Fe is used in cell division, photosynthesis, nitrogen fixation, and the formation and morphology of plants [9]. Deficiency in Fe often leads to interveinal chlorosis due to the reduction of chlorophyll production, and as the deficiency progresses, the entire leaf will become whitish-yellow and develop necrosis [10]. Mo is involved in several enzyme systems and in particular nitrate reductase, which is required for the reduction of nitrate, and nitrogenase that is involved in biological nitrogen fixation (BNF). Due to this interrelationship, deficiency symptoms of Mo are often similar to N deficiency symptoms with stunted growth and chlorosis occurring in legumes [11].

Toxic effects of heavy metals on growth, development and metabolism of different crop species.

Phytotoxic concentration of Zn has been reported to the result in decrease of growth, development, and metabolism, and it induces oxidative damage in various plant species such as *Phaseolus vulgaris* and *Brassica juncea* [12]. Excessive Zn concentration decreased the content of photosynthetic pigments such as chlorophyll a (Chl 𝑎) and chlorophyll b (Chl 𝑏) by interrupting the uptake and translocation of Mg and Fe into chloroplast of many crop species viz., bean [13], sorghum [14] and naked pumpkin [15] etc. Zn toxicity also causes the deficiency of Mn, Fe, and Cu in plant shoots, chlorosis in the young leaves and purplish-red color in leaves (ascribed to phosphorus deficiency) [16]. Cd is considered very toxic for plants inhibiting photosynthesis, and reducing root and shoot growth [17]. The roots are one of the target organs of Cd pollution, and root growth of crops such as wheat, maize, pumpkin, cucumber and garlic (*Allium sativum* L.) were severely inhibited [18]. Growth of vegetables such as cabbage [19], carrots [20], broccoli [21] and cucumbers [22] was inhibited under exposure to 10 mg/L Cd solution. Cd has been also observed to induce strong inhibition of chlorophylls biosynthesis in various crops such as soybean [23], faba bean [24], sorghum [25], cotton [26], barley [27] and maize [28], resulting in considerable decline of Chl a, Chl b and carotenoid concentration. Cd reduced nitrogen fixation, nodulation and primary ammonia assimilation in nodules of legumes, and such changes result in considerable reduction of their productivity due to ineffective nodulation and the lesser availability of nitrogen [29]. Furthermore, Cd toxicity has been also reported to cause inhibition of seed germination and root growth in chickpea [30] and barley [31], as well as decreases, root fresh weight and dry weight in faba bean [24] and maize [28].

The first physiological process affected by Cr is seed germination, and the tolerance of crop species to this metal is determined by the ability of a seed to germinate in medium containing Cr [32]. High levels of Cr in soil reduced germination up to 48% in the bush bean (Phaseolus vulgaris) [33]. The reduction in seed germination by Cr stress has been also reported in many other crop species such as *Medicago sativa* (23%), sugarcane (32–57%) and *L. perenne* [16]. Cr stress also affects photosynthesis in terms of Co2 fixation, electron transport, photophosphorylation, and enzyme activities [34]. Fozia et al. (2008) [35] assessing effects of Cr on growth attributes of sunflower (Helianthus annuus L.) ascribed the observed decrease in root length to cell cycle extension triggered by Cr toxicity. In addition, Cr interferes with the absorption and accumulation of number of the other metals or nutrients such as Fe, Ca, Mg, Mn, P and K in both aerial or root parts of plants, mostly leading to their reduced cellular or tissue concentration [36]. Pb exerts an adverse effect on morphology, growth and photosynthetic processes of plants, inhibits early seedlings growth in soybean, rice, maize, barley, tomato, egg plant, pigeonpea and certain legumes [37]. It also inhibited root and stem elongation as well as leaf expansion in Allium species, barley, *Sesamum indicum* and *Raphanus sativas* [16]. Pb treatment has been observed to reduce morphological parameters such as shoot/root length, shoot fresh and dry weight, and number of tillers in wheat [38]. Phytotoxicity study of Co in barley, oilseed rape, chickpea, and tomato has revealed its adverse effect on shoot growth and biomass [39]. Co toxicity decreased plant growth, nutrient content, biochemical content and antioxidant enzyme activities in radish and mungbean [40] [41]. Imtiyaz et al. (2014) [42] reported significant reduction of seed germination percentage, seedling growth, leaf area, root development and biomass production at high concentration of Co in soybean.

The toxic concentration of Ni in soil results in various physiological alterations and diverse toxicity symptoms such as chlorosis and necrosis in different plant species [43]. High uptake of Ni induced a decline in the water content of dicot and monocot plant species [44]. Kaveriammal and Subramani, (2015) [45] observed a significant reduction of root/shoot growth as well as fresh weight of the seedlings at 100 mg^.^L^-1^ Ni concentration in groundnut. Furthermore, Mn toxicity leading to necrotic brown spotting on leaves, petioles and stems as well as ‘‘crinkleleaf’’ are its common symptoms [46,47]. Roots exhibiting Mn toxicity are commonly brown in color and sometimes crack [48]. In addition, excess Mn inhibits synthesis of chlorophyll by blocking Fe-concerning process [49]. Kibra (2008) [50] recorded significant reduction in height, tiller number and panicle formation of rice plants growing on a soil contaminated with 1mgHg/kg. Kumar et al., (2016) [51] also reported reduction in germination percentage, radical and plumule lengths and seedling emergence percentage of wheat seeds subjected to HgCl2. The germination parameters like germination percentage, root and shoot length, fresh and dry weight of 10 days seedlings are significantly decreased with the increase in concentration of HgCl_2_ in pigeonpea [52]. Sharma et al. (2010) [53] working with spinach seeds (*Spinacia oleracea* L.) found a significant negative correlation between the root and shoot elongation with increasing Cu levels, which was associated with a noticeable depression in seedling fresh weight. Furthermore, Cu stress was observed to led decreased growth in terms of shoot length, fresh and dry weights as well as increased lipid peroxidation, deteriorated Chl b and carotenoids in pigeonpea, which in turn leads to reduced plant growth and metabolism [54].

References:

1. Ricachenevsky, F.K.; Menguer, P.K.; Sperotto, R.A.; Williams, L.E.; Fett, J.P. Roles of plant metal tolerance proteins (MTP) in metal storage and potential use in biofortification strategies. *Frontiers in plant science* **2013**, *4*, 144.
2. Cakmak, I. Tansley Review No. 111 Possible roles of zinc in protecting plant cells from damage by reactive oxygen species. *The New Phytologist* **2000**, *146*, 185–205.
3. Rengel, Z. Physiological mechanisms underlying differential nutrient efficiency of crop genotypes. *Mineral nutrition of crops: fundamental mechanisms implications* **1999**, 227-265.
4. Mahmood, T.; Islam, K. Response of rice seedlings to copper toxicity and acidity. *Journal of plant nutrition* **2006**, *29*, 943–957.
5. Havlin, J.L.; Beaton, J.D.; Tisdale, S.L.; Nelson, W.L. *Soil fertility and fertilizers: An introduction to nutrient management*; Pearson Prentice Hall Upper Saddle River, NJ: 2005; Vol. 515.
6. Gerendás, J.; Polacco, J.C.; Freyermuth, S.K.; Sattelmacher, B. Significance of nickel for plant growth and metabolism. *Journal of Plant Nutrition Soil Science* **1999**, *162*, 241–256.
7. Mengel, K.; Kirkby, E.A. Principles of plant nutrition. *Principles of plant nutrition.* **1978**.
8. Jacobsen, J.; Jasper, C. Diagnosis of Nutrient Deficiencies in Alfalfa and Wheat: Montana State University. *Extension Service* **1991**.
9. Alexander, C.R.; Smith, R.G.; Calder, F.D.; Schropp, S.J.; Windom, H.L. The historical record of metal enrichment in two Florida estuaries. *Estuaries* **1993**, *16*, 627–637.
10. Follett, R.H.; Westfall, D.G.; Croissant, R.L. *Utilization of animal manure as fertilizer*; Colorado State University, Cooperative Extension: 1992.
11. McCauley, A.; Jones, C.; Jacobsen, J. Soil pH and organic matter. *Nutrient management module* **2009**, *8*, 1–12.
12. Nagajyoti, P.C.; Lee, K.D.; Sreekanth, T. Heavy metals, occurrence and toxicity for plants: a review. *Environmental chemistry letters* **2010**, *8*, 199–216.
13. Vassilev, A.; Nikolova, A.; Koleva, L.; Lidon, F. Effects of excess Zn on growth and photosynthetic performance of young bean plants. *Journal of Phytology* **2011**.
14. Mirshekali, H.; Hadi, H.; Amirnia, R.; Khodaverdiloo, H. Effect of zinc toxicity on plant productivity, chlorophyll and Zn contents of sorghum (Sorghum bicolor) and common lambsquarter (Chenopodium album). *International Journal of Agriculture: Research Review* **2012**, *2*, 247–254.
15. Lalelou, F.S.; Shafagh-Kolvanagh, J.; Fateh, M. Effect of salinity on germination indexes of medicinal plant naked pumpkin (Cucurbita pepo). *International Journal of Agriculture Crop Sciences* **2013**, *5*, 1424.
16. Gill, M. Heavy metal stress in plants: a review. *Int J Adv Res* **2014**, *2*, 1043–1055.
17. Qian, H.; Li, J.; Sun, L.; Chen, W.; Sheng, G.D.; Liu, W.; Fu, Z. Combined effect of copper and cadmium on Chlorella vulgaris growth and photosynthesis-related gene transcription. *Aquatic toxicology* **2009**, *94*, 56–61.
18. Cheng, S. Effects of heavy metals on plants and resistance mechanisms. *Environmental Science Pollution Research* **2003**, *10*, 256–264.
19. Pandey, N.; Sharma, C.P. Effect of heavy metals Co2+, Ni2+ and Cd2+ on growth and metabolism of cabbage. *Plant Science* **2002**, *163*, 753–758, doi:https://doi.org/10.1016/S0168-9452(02)00210-8.
20. Salim, R.; Al-Subu, M.M.; Atallah, A. Effects of root and foliar treatments with lead, cadmium, and copper on the uptake distribution and growth of radish plants. *Environment International* **1993**, *19*, 393–404, doi:https://doi.org/10.1016/0160-4120(93)90130-A.
21. Mourato, M.P.; Moreira, I.N.; Leitão, I.; Pinto, F.R.; Sales, J.R.; Martins, L.L. Effect of Heavy Metals in Plants of the Genus Brassica. *International Journal of Molecular Sciences* **2015**, *16*, 17975.
22. Moral, R.; Pérez-Espinosa, A.; Pérez-Murcia, M.D. Cadmium accumulation and distribution in cucumber plant AU - Moreno-Caselles, J. *Journal of Plant Nutrition* **2000**, *23*, 243–250, doi:10.1080/01904160009382011.
23. Zirulnik, F.; Felici, C.E.; Almeida, C.A.; Baldo, F.; Alejandro, M.; Martinez, L.D.; Gomez, M.R.A. Analysis of Metal Profile in Soybean (Glycine max L.) after Cadmiun-induced Oxidative Damage. *Journal of Coastal Life Medicine* **2014**.
24. Piršelová, B.; Kuna, R.; Lukáč, P.; Havrlentová, M. Effect of cadmium on growth, photosynthetic pigments, iron and cadmium accumulation of Faba Bean (Vicia faba cv. Aštar). *Agriculture* **2016**, *62*, 72–79.
25. Da-lin, L.; Kai-qi, H.; Jing-jing, M.; Wei-wei, Q.; Xiu-ping, W.; Shu-pan, Z. Effects of cadmium on the growth and physiological characteristics of sorghum plants. *African Journal of Biotechnology* **2011**, *10*, 15770–15776.
26. Liu, L.; Chen, J.; Sun, H.; Zhang, Y.; Li, C. Effects of cadmium stress on growth and cadmium accumulation in cotton (Gossypium hirsutum L.) seedlings. *Cotton Science* **2014**, *5*, 466–470.
27. Gubrelay, U.; Agnihotri, R.K.; Singh, G.; Kaur, R.; Sharma, R. Effect of heavy metal Cd on some physiological and biochemical parameters of Barley (Hordeum vulgare L.). *International Journal of Agriculture Crop Sciences* **2013**, *5*, 2743.
28. Zahoor, A.; Lal, E.; Shukla, P.K.; Ramteke, P.W. Morphological and Biochemical Fluctuations in Maize Varieties Grown under Cadmium Stressed Environment. *VEGETOS* **2015**, 152.
29. Thakur, A.K.; Singh, K.J. Heavy metal Cd affecting nodulation and leghaemoglobin proteins in soybean and chickpea, BMR Microbiology. *J. Plant Agri. Res* **2014**.
30. Mondal, N.K.; Das, C.; Roy, S.; Datta, J.K.; Banerjee, A. Effect of varying cadmium Stress on chickpea (Cicer arietinum l) seedlings: An Ultrastructural study. *Annals of Environmental Science* **2013**, *7*.
31. Zhang, Y. Toxicity of heavy metals to hordeum vulgare. *Acta Scientiae Circumstantiae* **1997**, *17*, 199–205.
32. Peralta, J.; Gardea-Torresdey, J.; Tiemann, K.; Gomez, E.; Arteaga, S.; Rascon, E.; Parsons, J. Uptake and effects of five heavy metals on seed germination and plant growth in alfalfa (Medicago sativa L.). *Bulletin of Environmental Contamination toxicology* **2001**, *66*, 727–734.
33. Parr, P.D.; Taylor Jr, F.G. Germination and growth effects of hexavalent chromium in Orocol TL (a corrosion inhibitor) on Phaseolus vulgaris. In Proceedings of 1982 UCC-ND/GAT ENVIRONMENTAL PROTECTION SEMINAR; p. 24.
34. Clijsters, H.v.; Van Assche, F. Inhibition of photosynthesis by heavy metals. *Photosynthesis Research* **1985**, *7*, 31–40.
35. Fozia, A.; Muhammad, A.Z.; Muhammad, A.; Zafar, M.K. Effect of chromium on growth attributes in sunflower (Helianthus annuus L.). *Journal of Environmental Sciences* **2008**, *20*, 1475–1480.
36. Samantaray, S.; Rout, G.R.; Das, P. Role of chromium on plant growth and metabolism. *Acta Physiologiae Plantarum* **1998**, *20*, 201–212.
37. Kalaivanan, D.; Ganeshamurthy, A.N. Mechanisms of heavy metal toxicity in plants. In *Abiotic Stress Physiology of Horticultural Crops*, Springer: 2016; pp. 85-102.
38. Bhatti, K.; Anwar, S.; Nawaz, K.; Hussain, K.; Siddiqi, E.; Sharif, R.; Talat, A.; Khalid, A. Effect of heavy metal lead (Pb) stress of different concentration on wheat (Triticum aestivum L.). *Middle-East. Journal of Scientific Research* **2013**, *14*, 148–154.
39. Li, H.-F.; Gray, C.; Mico, C.; Zhao, F.-J.; McGrath, S.P. Phytotoxicity and bioavailability of cobalt to plants in a range of soils. *Chemosphere* **2009**, *75*, 979–986.
40. Jayakumar, K.; Vijayarengan, P. Alterations in the carbohydrate metabolism of Vigna mungo (L.) Hepper as affected by cobalt stress. *Envoinformatics* **2008**, *52*, 344–347.
41. Jayakumar, K.; Jaleel, C.A.; Vijayarengan, P. Changes in growth, biochemical constituents, and antioxidant potentials in radish (Raphanus sativus L.) under cobalt stress. *Turkish Journal of Biology* **2007**, *31*, 127–136.
42. Imtiyaz, S.; Agnihotri, R.K.; Ganie, S.A.; Sharma, R. Biochemical response of Glycine max (L.) Merr. to cobalt and lead stress. *Journal of Stress Physiology Biochemistry* **2014**, *10*, 259–272.
43. Rahman, H.; Sabreen, S.; Alam, S.; Kawai, S. Effects of nickel on growth and composition of metal micronutrients in barley plants grown in nutrient solution. *Journal of Plant Nutrition* **2005**, *28*, 393–404.
44. Fatemeh, G.; Reza, H.; Rashid, J.; Latifeh, P. Effects of Ni2+ toxicity on Hill reaction and membrane functionality in maize. *Journal of Stress Physiology Biochemistry* **2012**, *8*.
45. Kaveriammal, S.; Subramani, A. Variation in seed germination and early growth of groundnut (Arachis hypogaea L.) under nickel treatments. *International Journal of Environment Bioenergy* **2015**, *10*, 47–53.
46. Wu, S.h. Effect of manganese excess on the soybean plant cultivated under various growth conditions. *Journal of Plant. Nutrition* **1994**, *17*, 991–1003.
47. Bachman, G.; Miller, W. Iron chelate inducible iron/manganese toxicity in zonal geranium. *Journal of plant nutrition* **1995**, *18*, 1917–1929.
48. Foy, C.; Weil, R.; Coradetti, C. Differential manganese tolerances of cotton genotypes in nutrient solution. *Journal of plant nutrition* **1995**, *18*, 685–706.
49. Clairmont, K.B.; Hagar, W.G.; Davis, E.A. Manganese toxicity to chlorophyll synthesis in tobacco callus. *Plant physiology* **1986**, *80*, 291–293.
50. Kibra, M. Effects of mercury on some growth parameters of rice (Oryza sativa L.). *Soil Environment* **2008**, *27*, 23–28.
51. Kumar, M.; Pant, B.; Mondal, S.; Bose, B. Hydro and halo priming: influenced germination responses in wheat Var-HUW-468 under heavy metal stress. *Acta physiologiae plantarum* **2016**, *38*, 217.
52. Patnaik, A.; Mohanty, B. Toxic effect of mercury and cadmium on germination and seedling growth of Cajanus cajan L.(pigeon pea). *Annals of Biological Research* **2013**, *4*, 123–126.
53. Sharma, R.; Devi, S.; dan Dhyani, P. Comparative assessment of the toxic effects of copper and cypermethrin using seeds of Spinacia Oleracea L. plants. *Tropical Ecology* **2010**, *51*, 375–387.
54. Sirhindi, G.; Sharma, P.; Singh, A.; Kaur, H.; Mir, M. Alteration in photosynthetic pigments, osmolytes and antioxidants in imparting copper stress tolerance by exogenous jasmonic acid treatment in Cajanus cajan. *International Journal of Plant Physiology Biochemistry* **2015**, *7*, 30–39.
