# Supplementary material for: Role of Silicon in Mitigation of Heavy Metal Stresses in Crop Plants
Source: Plants (Basel). 2019 Mar 21;8(3):71. doi: 10.3390/plants8030071 (PMC6473438; doi:10.3390/plants8030071)
Supplement: Supplementary file 1 [file plants-08-00071-s001.zip › Supplementary Text2.docx]

Supplementary Text 2: Details of references provided in the Table 1, 2, and 3

References

1. Kibra, M. Effects of mercury on some growth parameters of rice (Oryza sativa L.). *Soil Environment* **2008**, *27*, 23–28.
2. Abedin, M.J.; Feldmann, J.; Meharg, A.A. Uptake kinetics of arsenic species in rice plants. *Plant physiology* **2002**, *128*, 1120–1128.
3. Li, B.; Quan-Wang, C.; Liu, H.; Li, H.-X.; Yang, J.; Song, W.-P.; Chen, L.; Zeng, M. Effects of Cd 2+ ions on root anatomical structure of four rice genotypes. *Journal of environmental biology* **2014**, *35*.
4. Bhatti, K.; Anwar, S.; Nawaz, K.; Hussain, K.; Siddiqi, E.; Sharif, R.; Talat, A.; Khalid, A. Effect of heavy metal lead (Pb) stress of different concentration on wheat (Triticum aestivum L.). *Middle-East Journal of Scientific Research* **2013**, *14*, 148–154.
5. Lamhamdi, M.; El Galiou, O.; Bakrim, A.; Nóvoa-Muñoz, J.C.; Arias-Estévez, M.; Aarab, A.; Lafont, R. Effect of lead stress on mineral content and growth of wheat (Triticum aestivum) and spinach (Spinacia oleracea) seedlings. *Saudi journal of biological sciences* **2013**, *20*, 29–36.
6. Gajewska, E.; Drobik, D.; Wielanek, M.; Sekulska-Nalewajko, J.; Gocławski, J.; Mazur, J.; Skłodowska, M. Alleviation of nickel toxicity in wheat (Triticum aestivum L.) seedlings by selenium supplementation. *Biological Letters* **2013**, *50*, 65–78.
7. Yourtchi, M.S.; Bayat, H.R. Effect of cadmium toxicity on growth, cadmium accumulation and macronutrient content of durum wheat (Dena CV.). *International Journal of Agriculture Crop Sciences* **2013**, *6*, 1199.
8. Panda, S.; Patra, H. Does chromium (III) produce oxidative damage in excised wheat leaves? *Journal of Plant Biology* **2000**, *27*, 105–110.
9. Pandolfini, T.; Gabbrielli, R.; Comparini, C. Nickel toxicity and peroxidase activity in seedlings of Triticum aestivum L. *Plant, Cell Environment* **1992**, *15*, 719–725.
10. Kleckerova, A.; Sobrova, P.; Krystofova, O.; Sochor, J.; Zitka, O.; Babula, P.; Adam, V.; Docekalova, H.; Kizek, R. Cadmium (II) and zinc (II) ions effects on maize plants revealed by spectroscopy and electrochemistry. *Int. J. Electrochem. Sci* **2011**, *6*, 6011–6031.
11. Fiala, R.; Kenderešová, L.; Syshchykov, D.; Martinka, M.; Repka, V.; Pavlovkin, J.; Čiamporová, M. Comparison of root growth and morphological responses to cadmium and nickel in two maize cultivars. *Modern Phytomorphology* **2013**, *3*, 131–137.
12. Wang, M.; Zou, J.; Duan, X.; Jiang, W.; Liu, D. Cadmium accumulation and its effects on metal uptake in maize (Zea mays L.). *Bioresource Technology* **2007**, *98*, 82–88.
13. Gubrelay, U.; Agnihotri, R.K.; Singh, G.; Kaur, R.; Sharma, R. Effect of heavy metal Cd on some physiological and biochemical parameters of Barley (Hordeum vulgare L.). *International Journal of Agriculture Crop Sciences* **2013**, *5*, 2743.
14. Žaltauskaitė, J.; Šliumpaitė, I. Evaluation of toxic effects and bioaccumulation of cadmium and copper in spring barley (Hordeum vulgare L.). *Environmental research, engineering management* **2013**, *64*, 51–58.
15. Kherbani, N.; Abdi, N.; Lounici, H. Effect of cadmium and zinc on growing barley. *Journal of Environmental Protection* **2015**, *6*, 160.
16. Kasim, W.A. Changes induced by copper and cadmium stress in the anatomy and grain yield of Sorghum bicolor (L.) Moench. *Int J Agri Biol* **2006**, *8*, 123–128.
17. Da-lin, L.; Kai-qi, H.; Jing-jing, M.; Wei-wei, Q.; Xiu-ping, W.; Shu-pan, Z. Effects of cadmium on the growth and physiological characteristics of sorghum plants. *African Journal of Biotechnology* **2011**, *10*, 15770–15776.
18. Moustakas, M.; Lanaras, T.; Symeonidis, L.; Karataglis, S. Growth and some photosynthetic characteristics of field grown Avena sativa under copper and lead stress. *Photosynthetica* **1994**.
19. Khan, R.; Srivastava, R.; Abdin, M.; Manzoor, N. Effect of soil contamination with heavy metals on soybean seed oil quality. *European Food Research Technology* **2013**, *236*, 707–714.
20. Zirulnik, F.; Felici, C.E.; Almeida, C.A.; Baldo, F.; Alejandro, M.; Martinez, L.D.; Gomez, M.R.A. Analysis of Metal Profile in Soybean (Glycine max L.) after Cadmiun-induced Oxidative Damage. *Journal of Coastal Life Medicine* **2014**.
21. Imtiyaz, S.; Agnihotri, R.K.; Ganie, S.A.; Sharma, R. Biochemical response of Glycine max (L.) Merr. to cobalt and lead stress. *Journal of Stress Physiology Biochemistry* **2014**, *10*, 259–272.
22. Thakur, A.K.; Singh, K.J. Heavy metal Cd affecting nodulation and leghaemoglobin proteins in soybean and chickpea, BMR Microbiology. *J. Plant Agri. Res* **2014**.
23. Cook, C.; Vardaka, E.; Lanaras, T. Concentrations of Cu, growth, and chlorophyll content of field-cultivated wheat growing in naturally enriched Cu soil. *Bulletin of environmental contamination toxicology* **1997**, *58*, 248–253.
24. Cakmak, I.; Marschner, H. Effect of zinc nutritional status on activities of superoxide radical and hydrogen peroxide scavenging enzymes in bean leaves. In *Plant Nutrition—from Genetic Engineering to Field Practice*, Springer: 1993; pp. 133-136.
25. Vassilev, A.; Nikolova, A.; Koleva, L.; Lidon, F. Effects of excess Zn on growth and photosynthetic performance of young bean plants. *Journal of Phytology* **2011**.
26. Mondal, N.K.; Das, C.; Roy, S.; Datta, J.K.; Banerjee, A. Effect of varying cadmium Stress on chickpea (Cicer arietinum l) seedlings: An Ultrastructural study. *Annals of Environmental Science* **2013**, *7*.
27. Anshula, S.; Gurpreet, S. Studies on the effect of Cu (II) ions on the antioxidant enzymes in chickpea (Cicer arietinum L.) cultivars. *Journal of Stress Physiology Biochemistry* **2013**, *9*.
28. Ali, B.; Hayat, S.; Hayat, Q.; Ahmad, A. Cobalt stress affects nitrogen metabolism, photosynthesis and antioxidant system in chickpea (Cicer arietinum L.). *Journal of Plant Interactions* **2010**, *5*, 223–231.
29. Sheoran, I.; Singal, H.; Singh, R. Effect of cadmium and nickel on photosynthesis and the enzymes of the photosynthetic carbon reduction cycle in pigeonpea (Cajanus cajan L.). *Photosynthesis Research* **1990**, *23*, 345–351.
30. Sujatha, B.; Priyadarshini, B. The effects of heavy metal on growth and mitochondrial changes in the Cajanus cajan. *Current Botany* **2013**, 27-32.
31. Patnaik, A.; Mohanty, B. Toxic effect of mercury and cadmium on germination and seedling growth of Cajanus cajan L.(pigeon pea). *Annals of Biological Research* **2013**, *4*, 123–126.
32. Arya, S.K.; Roy, B. Manganese induced changes in growth, chlorophyll content and antioxidants activity in seedlings of broad bean (Vicia faba L.). *Journal of Environmental Biology* **2011**, *32*, 707.
33. Piršelová, B.; Kuna, R.; Lukáč, P.; Havrlentová, M. Effect of cadmium on growth, photosynthetic pigments, iron and cadmium accumulation of Faba Bean (Vicia faba cv. Aštar). *Agriculture* **2016**, *62*, 72–79.
34. Doncheva, S.; Georgieva, K.; Vassileva, V.; Stoyanova, Z.; Popov, N.; Ignatov, G. Effects of succinate on manganese toxicity in pea plants. *Journal of plant nutrition* **2005**, *28*, 47–62.
35. Doncheva, S.; Stoynova, Z.; Velikova, V. Influence of succinate on zinc toxicity of pea plants. *Journal of plant nutrition* **2001**, *24*, 789–804.
36. Jayakumar, K.; Vijayarengan, P. Alterations in the carbohydrate metabolism of Vigna mungo (L.) Hepper as affected by cobalt stress. *Envoinformatics* **2008**, *52*, 344–347.
37. Marka, N.; Anil Kumar, S.; Rao, M. *Constitutive Effects of Distinct Heavy Metals (Cd, Pb and As) on Seed Germination and Physiological Characters of Groundnut (Arachis hypogaea L.)*; 2015; Vol. 3, pp. 959-970.
38. Gopal, R.; Sharma, Y. Effects of Chromium (VI) on Groundnut (Arachis Hypogeae L.) Metabolism. *J Plant Physiol Pathol 2* **2014**, *2*, 2.
39. Kaveriammal, S.; Subramani, A. Variation in seed germination and early growth of groundnut (Arachis hypogaea L.) under nickel treatments. *International Journal of Environment Bioenergy* **2015**, *10*, 47–53.
40. Liu, L.; Chen, J.; Sun, H.; Zhang, Y.; Li, C. Effects of cadmium stress on growth and cadmium accumulation in cotton (Gossypium hirsutum L.) seedlings. *Cotton Science* **2014**, *5*, 466–470.
41. Bharwana, S.; Ali, S.; Farooq, M.; Iqbal, N.; Abbas, F.; Ahmad, M. Alleviation of lead toxicity by silicon is related to elevated photosynthesis, antioxidant enzymes suppressed lead uptake and oxidative stress in cotton. *J. Bioremed. Biodeg* **2013**, *4*, 10.4172.
42. Carbonell-Barrachina, A.; Burlo, F.; Burgos-Hernandez, A.; Lopez, E.; Mataix, J. The influence of arsenite concentration on arsenic accumulation in tomato and bean plants. *Scientia Horticulturae* **1997**, *71*, 167–176.
43. Jayakumar, K.; Rajesh, M.; Baskaran, L.; Vijayarengan, P. Changes in nutritional metabolism of tomato (Lycopersicon esculantum Mill.) plants exposed to increasing concentration of cobalt chloride. *International Journal of Food Nutrition Safety* **2013**, *4*, 62–69.
44. Moral, R.; Gomez, I.; Pedreno, J.; Mataix, J. Absorption of Cr and effects on micronutrient content in tomato plant (Lycopersicum esculentum M). *Agrochimica* **1996**, *40*, 132–138.
45. Shekar, C.C.; Sammaiah, D.; Shasthree, T.; Reddy, K.J. Effect of mercury on tomato growth and yield attributes. *International Journal of Pharma Bio Sciences* **2011**, *2*, B358–B364.
46. Cox, M.; Bell, P.; Kovar, J. Differential tolerance of canola to arsenic when grown hydroponically or in soil. *Journal of Plant Nutrition* **1996**, *19*, 1599–1610.
47. John, R.; Ahmad, P.; Gadgil, K.; Sharma, S. Heavy metal toxicity: Effect on plant growth, biochemical parameters and metal accumulation by Brassica juncea L. *International Journal of Plant Production* **2012**, *3*, 65–76.
48. Jiang, W.; Liu, D.; Hou, W. Hyperaccumulation of cadmium by roots, bulbs and shoots of garlic (Allium sativum L.). *Bioresource Technology* **2001**, *76*, 9–13.
49. Nematshahi, N.; Lahouti, M.; Ganjeali, A. Accumulation of chromium and its effect on growth of (Allium cepa cv. Hybrid). *European Journal of Experimental Biology* **2012**, *2*.
50. Jayakumar, K.; Jaleel, C.A.; Vijayarengan, P. Changes in growth, biochemical constituents, and antioxidant potentials in radish (Raphanus sativus L.) under cobalt stress. *Turkish Journal of Biology* **2007**, *31*, 127–136.
51. Gu, H.-H.; Qiu, H.; Tian, T.; Zhan, S.-S.; Chaney, R.L.; Wang, S.-Z.; Tang, Y.-T.; Morel, J.-L.; Qiu, R.-L. Mitigation effects of silicon rich amendments on heavy metal accumulation in rice (Oryza sativa L.) planted on multi-metal contaminated acidic soil. *Chemosphere* **2011**, *83*, 1234–1240.
52. Seyfferth, A.L.; Fendorf, S. Silicate mineral impacts on the uptake and storage of arsenic and plant nutrients in rice (Oryza sativa L.). *Environmental science technology* **2012**, *46*, 13176–13183.
53. Liu, J.; Cai, H.; Mei, C.; Wang, M. Effects of nano-silicon and common silicon on lead uptake and translocation in two rice cultivars. *Frontiers of Environmental Science Engineering* **2015**, *9*, 905–911.
54. Nwugo, C.C.; Huerta, A.J. Silicon‐induced cadmium resistance in rice (Oryza sativa). *Journal of plant nutrition soil science* **2008**, *171*, 841–848.
55. Farooq, M.A.; Detterbeck, A.; Clemens, S.; Dietz, K.-J. Silicon-induced reversibility of cadmium toxicity in rice. *Journal of experimental botany* **2016**, *67*, 3573–3585.
56. Zhang, C.; Wang, L.; Nie, Q.; Zhang, W.; Zhang, F. Long-term effects of exogenous silicon on cadmium translocation and toxicity in rice (Oryza sativa L.). *Environmental Experimental Botany* **2008**, *62*, 300–307.
57. da Cunha, K.P.V.; do Nascimento, C.W.A. Silicon effects on metal tolerance and structural changes in maize (Zea mays L.) grown on a cadmium and zinc enriched soil. *Water, air, soil pollution* **2009**, *197*, 323.
58. Wang, Y.; Stass, A.; Horst, W.J. Apoplastic binding of aluminum is involved in silicon-induced amelioration of aluminum toxicity in maize. *Plant physiology* **2004**, *136*, 3762–3770.
59. Wang, L.; Wang, Y.; Chen, Q.; Cao, W.; Li, M.; Zhang, F. Silicon induced cadmium tolerance of rice seedlings. *Journal of plant nutrition* **2000**, *23*, 1397–1406.
60. Liang, Y.; Sun, W.; Zhu, Y.-G.; Christie, P. Mechanisms of silicon-mediated alleviation of abiotic stresses in higher plants: A review. *Environmental pollution* **2007**, *147*, 422–428.
61. Kaya, C.; Tuna, A.L.; Sonmez, O.; Ince, F.; Higgs, D. Mitigation effects of silicon on maize plants grown at high zinc. *Journal of plant nutrition* **2009**, *32*, 1788–1798.
62. Hussain, I.; Ashraf, M.A.; Rasheed, R.; Asghar, A.; Sajid, M.A.; Iqbal, M. Exogenous application of silicon at the boot stage decreases accumulation of cadmium in wheat (Triticum aestivum L.) grains. *Brazilian Journal of Botany* **2015**, *38*, 223–234.
63. Naeem, A.; Ghafoor, A.; Farooq, M. Suppression of cadmium concentration in wheat grains by silicon is related to its application rate and cadmium accumulating abilities of cultivars. *Journal of the Science of Food Agriculture* **2015**, *95*, 2467–2472.
64. Keller, C.; Rizwan, M.; Davidian, J.-C.; Pokrovsky, O.; Bovet, N.; Chaurand, P.; Meunier, J.-D. Effect of silicon on wheat seedlings (Triticum turgidum L.) grown in hydroponics and exposed to 0 to 30 µM Cu. *Planta* **2015**, *241*, 847–860.
65. Greger, M.; Landberg, T.; Vaculik, M.; Lux, A. Silicon influences nutrient status in plants. *Silicon in Agriculture* **2011**, 57.
66. Hammond, K.E.; Evans, D.E.; Hodson, M.J. Aluminium/silicon interactions in barley (Hordeum vulgare L.) seedlings. *Plant soil* **1995**, *173*, 89–95.
67. Ali, S.; Farooq, M.A.; Yasmeen, T.; Hussain, S.; Arif, M.S.; Abbas, F.; Bharwana, S.A.; Zhang, G. The influence of silicon on barley growth, photosynthesis and ultra-structure under chromium stress. *Ecotoxicology environmental safety* **2013**, *89*, 66–72.
68. Shi, Q.; Bao, Z.; Zhu, Z.; He, Y.; Qian, Q.; Yu, J. Silicon-mediated alleviation of Mn toxicity in Cucumis sativus in relation to activities of superoxide dismutase and ascorbate peroxidase. *Phytochemistry* **2005**, *66*, 1551–1559.
69. Dragišić Maksimović, J.; Mojović, M.; Maksimović, V.; Römheld, V.; Nikolic, M. Silicon ameliorates manganese toxicity in cucumber by decreasing hydroxyl radical accumulation in the leaf apoplast. *Journal of experimental botany* **2012**, *63*, 2411–2420.
70. Horst, W.J.; Fecht, M.; Naumann, A.; Wissemeier, A.H.; Maier, P. Physiology of manganese toxicity and tolerance in Vigna unguiculata (L.) Walp. *Journal of Plant Nutrition Soil Science* **1999**, *162*, 263–274.
71. Iwasaki, K.; Maier, P.; Fecht, M.; Horst, W.J. Leaf apoplastic silicon enhances manganese tolerance of cowpea (Vigna unguiculata). *Journal of Plant Physiology* **2002**, *159*, 167–173.
72. Shi, G.; Cai, Q.; Liu, C.; Wu, L. Silicon alleviates cadmium toxicity in peanut plants in relation to cadmium distribution and stimulation of antioxidative enzymes. *Plant Growth Regulation* **2010**, *61*, 45–52.
73. Li, J.; Leisner, S.M.; Frantz, J. Alleviation of copper toxicity in Arabidopsis thaliana by silicon addition to hydroponic solutions. *Journal of the American Society for Horticultural Science* **2008**, *133*, 670–677.
74. Vatehová, Z.; Kollárová, K.; Zelko, I.; Richterová-Kučerová, D.; Bujdoš, M.; Lišková, D. Interaction of silicon and cadmium in Brassica juncea and Brassica napus. *Biologia* **2012**, *67*, 498–504.
75. Neumann, D.; zur Nieden, U.; Schwieger, W.; Leopold, I.; Lichtenberger, O. Heavy metal tolerance of Minuartia verna. *Journal of plant physiology* **1997**, *151*, 101–108.
76. Neumann, D.; Zur Nieden, U. Silicon and heavy metal tolerance of higher plants. *Phytochemistry* **2001**, *56*, 685–692.
77. Song, A.; Li, Z.; Zhang, J.; Xue, G.; Fan, F.; Liang, Y. Silicon-enhanced resistance to cadmium toxicity in Brassica chinensis L. is attributed to Si-suppressed cadmium uptake and transport and Si-enhanced antioxidant defense capacity. *Journal of Hazardous Materials* **2009**, *172*, 74–83.
78. Farooq, M.A.; Ali, S.; Hameed, A.; Ishaque, W.; Mahmood, K.; Iqbal, Z. Alleviation of cadmium toxicity by silicon is related to elevated photosynthesis, antioxidant enzymes; suppressed cadmium uptake and oxidative stress in cotton. *Ecotoxicology environmental safety* **2013**, *96*, 242–249.
79. Mitani, N.; Yamaji, N.; Ma, J.F. Identification of maize silicon influx transporters. *Plant Cell Physiology* **2008**, *50*, 5–12.
80. Mitani, N.; Chiba, Y.; Yamaji, N.; Ma, J.F. Identification and characterization of maize and barley Lsi2-like silicon efflux transporters reveals a distinct silicon uptake system from that in rice. *The Plant Cell* **2009**, *21*, 2133–2142.
81. Ma, J.F.; Tamai, K.; Yamaji, N.; Mitani, N.; Konishi, S.; Katsuhara, M.; Ishiguro, M.; Murata, Y.; Yano, M. A silicon transporter in rice. *Nature* **2006**, *440*, 688.
82. Ma, J.F.; Yamaji, N.; Mitani, N.; Tamai, K.; Konishi, S.; Fujiwara, T.; Katsuhara, M.; Yano, M. An efflux transporter of silicon in rice. *Nature* **2007**, *448*, 209.
83. Yamaji, N.; Mitatni, N.; Ma, J.F. A transporter regulating silicon distribution in rice shoots. *The Plant Cell* **2008**, *20*, 1381–1389.
84. Yamaji, N.; Sakurai, G.; Mitani-Ueno, N.; Ma, J.F. Orchestration of three transporters and distinct vascular structures in node for intervascular transfer of silicon in rice. *Proceedings of the National Academy of Sciences* **2015**, *112*, 11401–11406.
85. Chiba, Y.; Mitani, N.; Yamaji, N.; Ma, J.F. HvLsi1 is a silicon influx transporter in barley. *The Plant Journal* **2009**, *57*, 810–818.
86. Yamaji, N.; Chiba, Y.; Mitani-Ueno, N.; Ma, J.F. Functional characterization of a silicon transporter gene implicated in Si distribution in barley. *Plant physiology* **2012**, pp. 112.204578.
87. Deshmukh, R.K.; Vivancos, J.; Guérin, V.; Sonah, H.; Labbé, C.; Belzile, F.; Bélanger, R.R. Identification and functional characterization of silicon transporters in soybean using comparative genomics of major intrinsic proteins in Arabidopsis and rice. *Plant molecular biology* **2013**, *83*, 303–315.
88. Montpetit, J.; Vivancos, J.; Mitani-Ueno, N.; Yamaji, N.; Rémus-Borel, W.; Belzile, F.; Ma, J.F.; Bélanger, R.R. Cloning, functional characterization and heterologous expression of TaLsi1, a wheat silicon transporter gene. *Plant molecular biology* **2012**, *79*, 35–46.
89. Mitani-Ueno, N.; Yamaji, N.; Ma, J.F. Silicon efflux transporters isolated from two pumpkin cultivars contrasting in Si uptake. *Plant signaling behavior* **2011**, *6*, 991–994.
90. Grégoire, C.; Rémus‐Borel, W.; Vivancos, J.; Labbé, C.; Belzile, F.; Bélanger, R.R. Discovery of a multigene family of aquaporin silicon transporters in the primitive plant Equisetum arvense. *The Plant Journal* **2012**, *72*, 320–330.
91. Vivancos, J.; Deshmukh, R.; Grégoire, C.; Rémus-Borel, W.; Belzile, F.; Bélanger, R.R. Identification and characterization of silicon efflux transporters in horsetail (Equisetum arvense). *Journal of plant physiology* **2016**, *200*, 82–89.
92. Vulavala, V.K.; Elbaum, R.; Yermiyahu, U.; Fogelman, E.; Kumar, A.; Ginzberg, I. Silicon fertilization of potato: Expression of putative transporters and tuber skin quality. *Planta* **2016**, *243*, 217–229.
93. Deshmukh, R.K.; Vivancos, J.; Ramakrishnan, G.; Guérin, V.; Carpentier, G.; Sonah, H.; Labbé, C.; Isenring, P.; Belzile, F.J.; Bélanger, R.R. A precise spacing between the NPA domains of aquaporins is essential for silicon permeability in plants. *The Plant Journal* **2015**, *83*, 489–500.
