# Supplementary material for: Role of Silicon in Mitigation of Heavy Metal Stresses in Crop Plants
Source: Plants (Basel). 2019 Mar 21;8(3):71. doi: 10.3390/plants8030071 (PMC6473438; doi:10.3390/plants8030071)
Supplement: Supplementary file 1 [file plants-08-00071-s001.zip › Table S1.docx]

**Table S1** Details of silicon transporter genes from different plant species validated using oocyte assay or transgenic approaches.

| S.No | Plant Species | Si-transporter gene | Assay used | References |
| --- | --- | --- | --- | --- |
| 1 | Rice | *Lsi1(OsNIP2;1)* | Oocyte& Transgenic | [1] |
| 2 |  |  |  |  |
| 3 |  | *OsLsi2* | Oocyte& Transgenic | [2] |
| 4 | Barley | *HvLsi1* | Oocyte | [3] |
| 5 |  | *HvLsi2* | Oocyte & Transgenic | [4] |
| 6 | Maize | *ZmLsi2* | Oocyte & Transgenic | [5] |
| 7 |  | *ZmLsi1 (ZmNIP2;1), ZmLsi6* | Oocyte | [5] |
| 8 | Wheat | *TaLsi1 (TaNIP2;1)* | Transgenic | [6] |
| 9 | Soybean | *GmNIP2-1,GmNIP2-2* | Oocyte& Transgenic | [7] |
| 10 | Pumpkin | *CmLsi1 (CmNIP2-1)* | Oocyte & Transgenic | [8] |
| 11 | Tomato | *SlNIP2-1(Mutant)* | Oocyte | [9] |
| 12 | Poplar | *PtNIP2-1* | Oocyte | [9] |
| 13 | Horsetail | *EaNIP3;1, EaNIP3;3, EaNIP3;4* | Oocyte | [10] |
| 14 | Horsetail | *EaNIP3;1, EaNIP3;3 and EaNIP3;4* | Oocyte | [10] |
| 15 |  | *EaNIP3;1* | Transgenic | [10] |
| 16 |  | *EaLsi2* | Oocyte | [11] |

References

1. Ma, J.F.; Tamai, K.; Yamaji, N.; Mitani, N.; Konishi, S.; Katsuhara, M.; Ishiguro, M.; Murata, Y.; Yano, M. A silicon transporter in rice. *Nature* **2006**, *440*, 688.
2. Ma, J.F.; Yamaji, N.; Mitani, N.; Tamai, K.; Konishi, S.; Fujiwara, T.; Katsuhara, M.; Yano, M. An efflux transporter of silicon in rice. *Nature* **2007**, *448*, 209.
3. Naeem, A.; Ghafoor, A.; Farooq, M. Suppression of cadmium concentration in wheat grains by silicon is related to its application rate and cadmium accumulating abilities of cultivars. *Journal of the Science of Food Agriculture* **2015**, *95*, 2467–2472.
4. Mitani, N.; Chiba, Y.; Yamaji, N.; Ma, J.F. Identification and characterization of maize and barley Lsi2-like silicon efflux transporters reveals a distinct silicon uptake system from that in rice. *The Plant Cell* **2009**, *21*, 2133–2142.
5. Mitani, N.; Yamaji, N.; Ma, J.F. Identification of maize silicon influx transporters. *Plant Cell Physiology* **2008**, *50*, 5–12.
6. Montpetit, J.; Vivancos, J.; Mitani-Ueno, N.; Yamaji, N.; Rémus-Borel, W.; Belzile, F.; Ma, J.F.; Bélanger, R.R. Cloning, functional characterization and heterologous expression of TaLsi1, a wheat silicon transporter gene. *Plant molecular biology* **2012**, *79*, 35–46.
7. Deshmukh, R.K.; Vivancos, J.; Guérin, V.; Sonah, H.; Labbé, C.; Belzile, F.; Bélanger, R.R. Identification and functional characterization of silicon transporters in soybean using comparative genomics of major intrinsic proteins in Arabidopsis and rice. *Plant molecular biology* **2013**, *83*, 303–315.
8. Mitani-Ueno, N.; Yamaji, N.; Ma, J.F. Silicon efflux transporters isolated from two pumpkin cultivars contrasting in Si uptake. *Plant signaling behavior* **2011**, *6*, 991–994.
9. Deshmukh, R.K.; Vivancos, J.; Ramakrishnan, G.; Guérin, V.; Carpentier, G.; Sonah, H.; Labbé, C.; Isenring, P.; Belzile, F.J.; Bélanger, R.R. A precise spacing between the NPA domains of aquaporins is essential for silicon permeability in plants. *The Plant Journal* **2015**, *83*, 489–500.
10. Grégoire, C.; Rémus‐Borel, W.; Vivancos, J.; Labbé, C.; Belzile, F.; Bélanger, R.R. Discovery of a multigene family of aquaporin silicon transporters in the primitive plant Equisetum arvense. *The Plant Journal* **2012**, *72*, 320–330.
11. Vivancos, J.; Deshmukh, R.; Grégoire, C.; Rémus-Borel, W.; Belzile, F.; Bélanger, R.R. Identification and characterization of silicon efflux transporters in horsetail (Equisetum arvense). *Journal of plant physiology* **2016**, *200*, 82–89.
